# Effect of Ion Irradiation Introduced by Focused Ion-Beam Milling on the Mechanical Behaviour of Sub-Micron-Sized Samples

**DOI:** 10.1038/s41598-020-66564-y

**Published:** 2020-06-25

**Authors:** Jinqiao Liu, Ranming Niu, Ji Gu, Matthew Cabral, Min Song, Xiaozhou Liao

**Affiliations:** 1 School of Aerospace, Mechanical & Mechatronic Engineering, The University of Sydney, Sydney, NSW 2006 Australia; 2 State Key Laboratory of Powder Metallurgy, Central South University, Changsha, 410083 China

**Keywords:** Nanoscale materials, Transmission electron microscopy, Metals and alloys

## Abstract

The development of xenon plasma focused ion-beam (Xe^+^ PFIB) milling technique enables site-specific sample preparation with milling rates several times larger than the conventional gallium focused ion-beam (Ga^+^ FIB) technique. As such, the effect of higher beam currents and the heavier ions utilized in the Xe^+^ PFIB system is of particular importance when investigating material properties. To investigate potential artifacts resulting from these new parameters, a comparative study is performed on transmission electron microscopy (TEM) samples prepared via Xe^+^ PFIB and Ga^+^ FIB systems. Utilizing samples prepared with each system, the mechanical properties of CrMnFeCoNi high-entropy alloy (HEA) samples are evaluated with *in situ* tensile straining TEM studies. The results show that HEA samples prepared by Xe^+^ PFIB present better ductility but lower strength than those prepared by Ga^+^ FIB. This is due to the small ion-irradiated volumes and the insignificant alloying effect brought by Xe irradiation. Overall, these results demonstrate that Xe^+^ PFIB systems allow for a more efficient material removal rate while imparting less damage to HEAs than conventional Ga^+^ FIB systems.

## Introduction

The rapid development of micro-electromechanical systems (MEMS) and nano-electromechanical systems (NEMS), which utilize materials at the micron scale and below, has resulted in a growing number of potential applications in electronic devices^1^. Mechanical properties are of particular importance for applications in M/NEMS as efforts seek to improve the functionality and reliability of advanced electronic devices. Continuing efforts have focused on understanding how the mechanical properties of these materials change with decreasing dimensions^2–6^. To facilitate this understanding, *in situ* straining transmission electron microscopy (TEM) is commonly used to test the mechanical properties^7–10^ and observe deformation mechanisms^11–17^ of small-sized samples. *In situ* straining TEM allows for simultaneous structural characterisation and mechanical property testing^13,15,18^, providing opportunities for building direct relationships between microstructure, deformation mechanisms, and mechanical properties of small-sized materials.

Sample preparation is of particular importance when studying small-sized materials in the TEM^19^. Traditionally, these TEM samples are prepared using a focused ion-beam (FIB) with a gallium ion (Ga^+^) source to thin samples from bulk to ~100 nm^20–26^. Despite technological advances, the material removal rates of Ga^+^ FIB systems have remained too low for researchers hoping to increase sample preparation efficiency^27^. To help facilitate more efficient sample preparation, researchers have developed FIB systems with alternative ion sources such as the Xe^+^ plasma FIB (Xe^+^ PFIB)^27^. As an alternative to Ga^+^ ions, Xe^+^ PFIB systems utilize inert Xe gas as the milling media resulting in material removal rates around six times larger than for Ga^+^ mills^27^, which enables the preparation of samples with larger dimensions. On the other hand, Xe^+^ PFIB induces a thinner amorphous layer on the sample surface compared to the Ga^+^ source^20,27,28^. Further, the low reactivity of the chemically inert Xe gas has enabled sample preparation of materials that are sensitive to many other types of ions^20,28^.

Sample preparation utilizing FIB techniques unavoidably introduces ion irradiation in materials that may potentially alter the microstructures and consequently the mechanical properties of samples^5,6,22,29–33^. Experiments demonstrate that Ga^+^ irradiation results in amorphization of a material surface^5,6,30,34^ which in turn increases their strength^30^ and hardness^5^. Additionally, ion penetration into materials will also introduce defects that may affect mechanical properties. For example, He^+^ irradiation tends to induce nanobubbles^31,32,35–39^ that enhance the ductility^32^ and sometimes even leads to superelasticity^37^. Some studies have explored the effects of Ga^+^ and He^+^ irradiation on the microstructures and mechanical properties of materials^5,30–32,34–39^. However, little effort has been made to understand how Xe^+^ affects the microstructures and mechanical properties of sub-micron-sized samples. Given the advantages of the PFIB technique, an investigation on the effects of Xe^+^ irradiation is essential for obtaining credible mechanical properties of prepared samples.

In this paper, we used the equiatomic CrMnFeCoNi HEA^40–42^ as the model material to explore the effects of ion milling on the characterisation of small-sized materials. High-entropy alloys (HEAs) have attracted considerable interests in recent years due to their superior mechanical properties and potential structural applications^43^. The CrMnFeCoNi HEA is one of the most promising HEAs that exhibits an excellent combination of strength and ductility especially at cryogenic temperatures^40–42,44^ as well as potential technological relevance for M/NEMS applications. The microstructure and mechanical properties of both Ga^+^ and Xe^+^ prepared small-sized dog-bone shaped HEA samples were investigated and compared by quantitative *in situ* straining TEM.

## Methods

The CrMnFeCoNi HEA with an equal atomic ratio for all five elements utilized in this work was prepared by arc-melting of a mixture of pure metals (purity > 99.99 wt.%) in a Ti-gettered high-purity argon atmosphere. Details of the alloy preparation can be found in ref. ^25^. Energy dispersive x-ray spectroscopy (EDS) elemental mapping was conducted in a Zeiss^®^ EVO 50 scanning electron microscope (SEM). The results show homogeneous chemical composition distribution, excluding any potential effect of chemical inhomogeneity on experimental results. A square piece with an edge length of ~10 mm and a thickness of ~3 mm was cut from a bulk sample, polished using silicon carbide (SiC) grinding paper followed by colloidal silica suspension until no obvious scratches were visible under an optical microscope. Electron backscatter diffraction (EBSD) was utilized to identify a large grain of ~130 μm in width with its surface perpendicular to a <001> zone axis. A lamella with dimensions of 100 × 7 × 4 μm^3^ was cut from the grain and lifted out with an FEI^®^ Helios G4 UXe dual-beam PFIB SEM. This lamella was utilized to prepare multiple *in situ* samples to ensure that all single-crystalline dog-bone shaped samples were of similar dimensions and the same crystallographic orientation. Dog-bone shape patterning of two sets of samples was conducted using FEI^®^ Helios G4 UXe dual-beam PFIB SEM and Zeiss^®^ Auriga Ga^+^ FIB SEM, respectively, with relevant sample preparation parameters presented in Table 1. The pre-tilted angles and beam currents were chosen based on a series of trial thinning tests to minimise taper angles and irradiation damage to samples. There was slight variation in current density of FIB and PFIB due to the design of the two microscopes, but the minor difference can be compensated by adjusting the aperture size^45^. Thermal effects due to heating during FIB milling contribute little impact to conductive materials^46^. Xe^+^ PFIB has a larger collision energy than Ga^+^ FIB due to the higher atomic number of Xe, which in turn contributes to the high milling rate of Xe^+^ PFIB. Given the above conditions, it is reasonable to assume that with identical processing parameters and similar Gaussian beam profiles, the only variable between the two sets of samples was the ion source used for sample preparation. The prepared dog-bone shaped samples were of gauge dimensions ~1,500 × 300 × 60 nm^3^ as determined via SEM utilizing a 5 kV electron beam with a beam current of 1.8 μA. Previous work has indicated that the electron beam has no detectable effect on the mechanical properties of metallic materials, which is different from materials with covalent or ionic bonds^47^. In total, three samples were prepared using each ion source with a >7 μm gap between neighbouring dog-bone shaped samples to avoid possible redeposition during ion milling. A wall was also utilized to separate the Xe-prepared samples from Ga-prepared samples to minimise contamination of samples from the other ion source.

**Table 1:** Parameters used in Xe^+^ PFIB and Ga^+^ FIB milling.

Technique		Xe^+^ PFIB	Ga^+^ FIB
Processing Stage			
Rough Milling	Equipment	Xe^+^ PFIB SEM	Xe^+^ PFIB SEM
	Current	30 kV, 1 nA	30 kV, 1 nA
Precise Patterning	Equipment	Xe^+^ PFIB SEM	Ga^+^ FIB SEM
	Current	30 kV, 30 pA	30 kV, 25 pA
	Pre-tilted angle	±2.3º	±2.2º
Polishing	Equipment	Xe^+^ PFIB SEM	Ga^+^ FIB SEM
	Current	5 kV, 30 pA	5 kV, 50 pA

**Figure 1:**
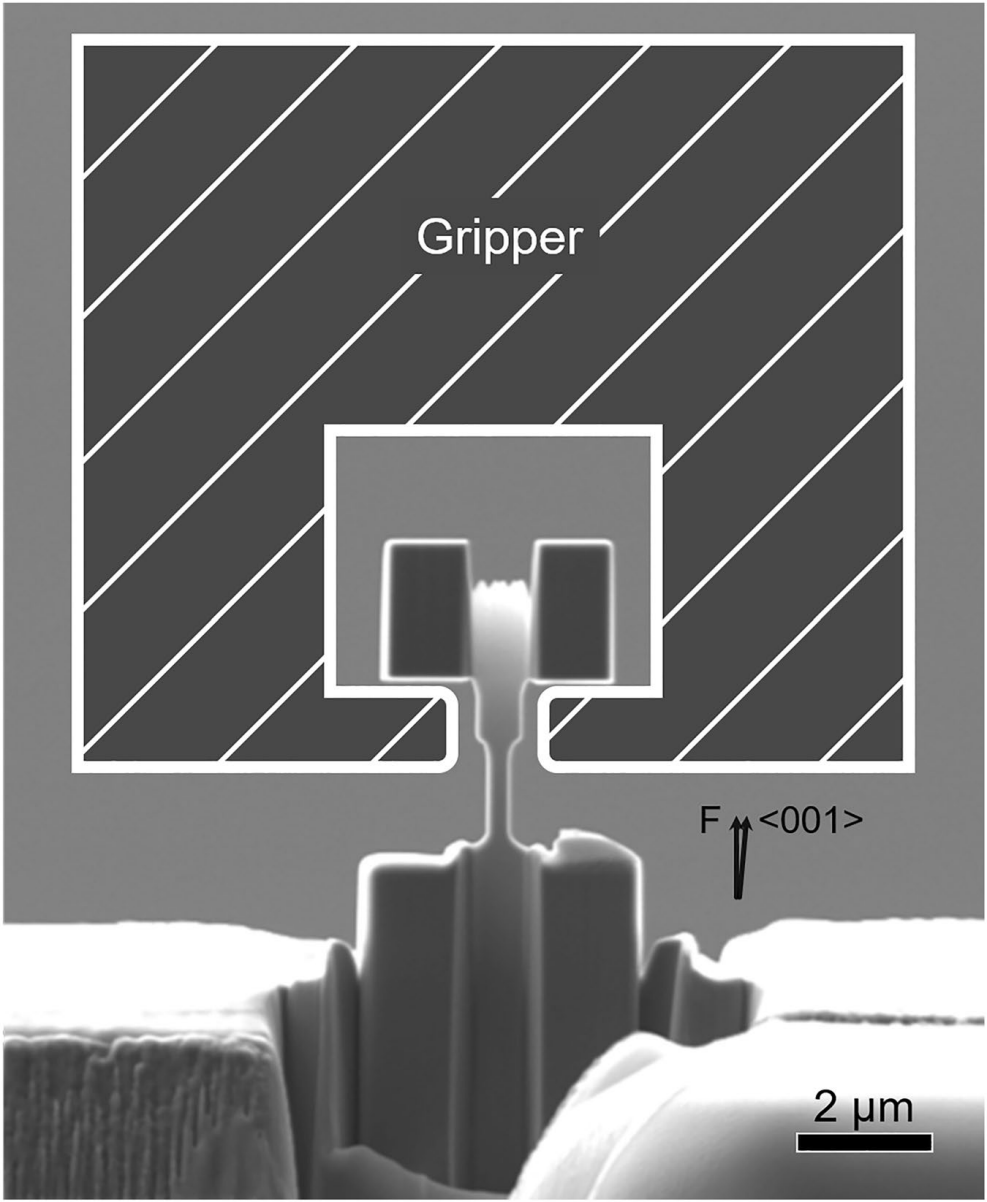
SEM image of a < 001 > -oriented single crystalline CrMnFeCoNi dog-bone shaped tensile sample. A schematic illustration of the tensile diamond gripper is superimposed on the image. The two arrows indicate the tensile loading direction (F) and a <001> direction of the specimen.

SEM and EBSD imaging were conducted using FEI^®^ Helios G4 UXe dual-beam PFIB SEM. *In situ* TEM tensile experiments and detailed structural characterization was carried out using a JEOL 2100 TEM operated at 200 keV. The tensile experiments were performed using a Hysitron PI95 Picoindenter^®^ specimen holder with a displacement control rate of 2 nm/s, which is equivalent to a strain rate of 10^−3^ s^−1^. Tensile deformation was achieved by pulling the end of one sample with a homemade diamond tensile gripper. Figure 1 shows an SEM image of a tensile dog-bone shaped sample with a schematic of the tensile specimen gripper. Selected area electron diffraction patterns indicated that the sample axial direction or the tensile loading direction was ~2° away from an exact <001> orientation.

## Results and Discussion

**Figure 2:**
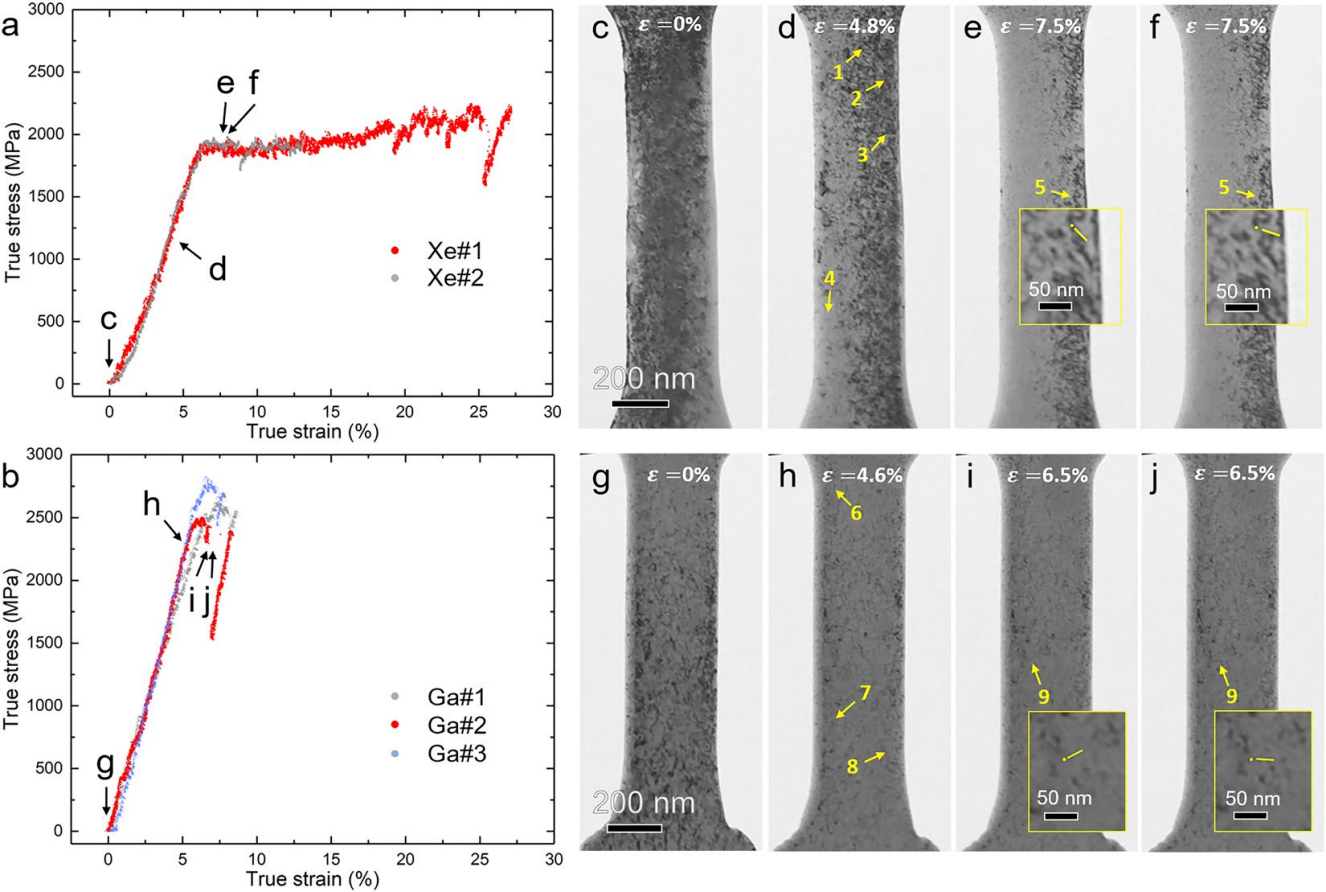
True stress–strain curves of the FIB/PFIB-fabricated samples and sequential snapshot TEM images extracted from *in situ* tensile test videos. (**a**,**b**) show true stress–strain curves of Xe^+^ fabricated and Ga^+^ fabricated samples, respectively. (**c–f**) present snapshot images of the Xe#1 sample at strain values marked by c–f in (**a**). (**g–j**) display snapshot images of the Ga#2 sample at strain values indicated using letters g–j in (**b**). Some defects in the snapshot images are marked with digits 1 to 9. Defects 5 and 9 are further magnified in (**e–f**) and (**i**–**j**), respectively.

The load–displacement was recorded during *in situ* tensile experiments then converted to true stress–strain curves. True stress–strain curves for two Xe^+^ fabricated samples and three Ga^+^ fabricated samples are shown in Fig. 2a,b, respectively. For post-mortem TEM observation, the first Xe^+^ fabricated sample (Xe#1) and the third Ga^+^ fabricated sample (Ga#3) were not strained to fracture. The Xe^+^ fabricated samples had a yield strength of ~1.9 GPa and a ductility of >20% (not yet fractured at ~20% for sample Xe#1), while the Ga^+^ fabricated samples exhibited a high yield strength of ~2.4 GPa but a significantly low ductility of ~3%. Note that bulk coarse-grained CrMnFeCoNi high-entropy alloys are typically very ductile at room temperature with a ductility between 40% – 60% but their yield strength is in the range of only ~170 MPa (grain size ~155 μm) – ~400 MPa (grain size ~6 μm)^40–43,48,49^. The significantly increased yield strength and the relatively large elastic strain of the as-prepared Xe^+^ fabricated and Ga^+^ fabricated samples were mainly the result of small-size effects on mechanical properties^7,50,51^. However, the consistency of the mechanical behaviour of the samples and the large difference in the strength and ductility between the Xe^+^ fabricated and Ga^+^ fabricated samples indicate that the ion sources used in sample preparation significantly affected the mechanical properties of the resulting small-sized samples. On the other hand, further investigations are needed to determine which ion source produces samples that possess mechanical properties closer to the intrinsic properties of the material, as either preservation of ductility^3,52^ or ductility-to-brittle transition^53^ may occur as dimension decreases.

To understand the reasons for the distinct mechanical properties of the Xe^+^ fabricated and Ga^+^ fabricated samples, detailed microstructural characterisation was conducted. Figure 2c–j present snapshot TEM images from videos (see Supplementary Information) acquired during the tensile straining processes of sample Xe#1 (Fig. 2c–f) and sample Ga#2 (Fig. 2g–j). Dislocations were observed before straining in both samples (Fig. 2c,g). The true stress–strain data in Fig. 2a,b of the two samples presented serrated pre-yielding curves, or microplasticity^54^. The previous research^55^ suggests that this phenomenon is caused by the “cleaning-up of pre-existing dislocations” through the motion and disappearance at the surface of ion-irradiation-induced dislocations, which exist at shallow surface regions of the samples at low-stress values and that this process imposes perturbations to the linear stress–strain curves at early stages of the deformation. Local dislocation motion during the “elastic” deformation process at locations annotated by digits 1–4 and 6–8 in Fig. 2d,h were observed at true strain values of 4.8% and 4.6% for samples Xe#1 and Ga#2, respectively (see Supplementary Information for videos). Yielding occurred only when the stress was large enough to trigger large dislocation motion.

Following yielding, further deformation for all samples occurred mainly via the single-arm dislocation source-controlled plasticity as large quantities of single-arm dislocation sources were seen active and operating (see Supplementary Information for videos), which is consistent with the source truncation mechanism^56,57^. An example of a single-arm dislocation, which is annotated by 5, in sample Xe#1 is shown in Fig. 2e,f. A magnified image of the dislocation is shown in the inset of Fig. 2e,f where the yellow dots indicate the pinned end of the dislocation and the short yellow lines show the instantaneous positions of the dislocation line (arm). The difference in the short line positions in Fig. 2e,f suggest the motion of the dislocation. Another example of similar dislocation activities is observed in sample Ga#2, as shown in Fig. 2i,j, in which a single-arm dislocation is marked by 9 and its magnified images are presented in the inset.

Although all samples share the same plastic deformation mechanism, the number of dislocation activities in the two types of samples differ. The Ga^+^ fabricated samples exhibited fewer dislocation activities than the Xe^+^ fabricated samples (see Supplementary Information for videos), indicating that it is more difficult to activate dislocations in Ga^+^ fabricated samples, which leads to higher strength but poorer ductility compared to Xe^+^ fabricated samples. Ion implantation of impurity elements during FIB processing likely contributes to the observed dislocation behaviour. It is well-known that impurities in materials exert a locking effect on dislocation activities^58–60^, reducing the mobility of dislocations and naturally the number of activated dislocation sources. The strength of this locking effect depends on the concentration and the type of impurity elements^58^. In our study, the concentration of impurity elements should not contribute significantly to the variation in the mechanical properties of the two types of samples as the concentration of both elements were too low, and the EDS analysis was not capable of capturing the minor difference in the concentration between Xe^+^ and Ga^+^ ion penetration. Therefore, the type of impurity elements is likely the major contributor to the difference in the locking effect. Different types of impurity elements exert different strength of locking effect due to differences in their electroactivity and size misfits with base atoms^58^. For example, it has been reported that phosphorus is more effective than oxygen at locking dislocation activities in silicon crystals because phosphorus has a higher electroactivity and similar size to silicon atoms than oxygen. In turn, this results in stronger electrostatic interactions with acceptor sites at dislocation cores^58^. Similarly, because Ga^+^ is more electroactive and has a similar radius to the elements in the high-entropy alloy than Xe^+^ plasma, it is reasonable to expect that implanted Ga^+^ ions exert a stronger locking effect on dislocations than Xe^+^ ions, and this accounts for the greater difficulty to activate the dislocation sources of Ga^+^ fabricated samples.

**Figure 3:**
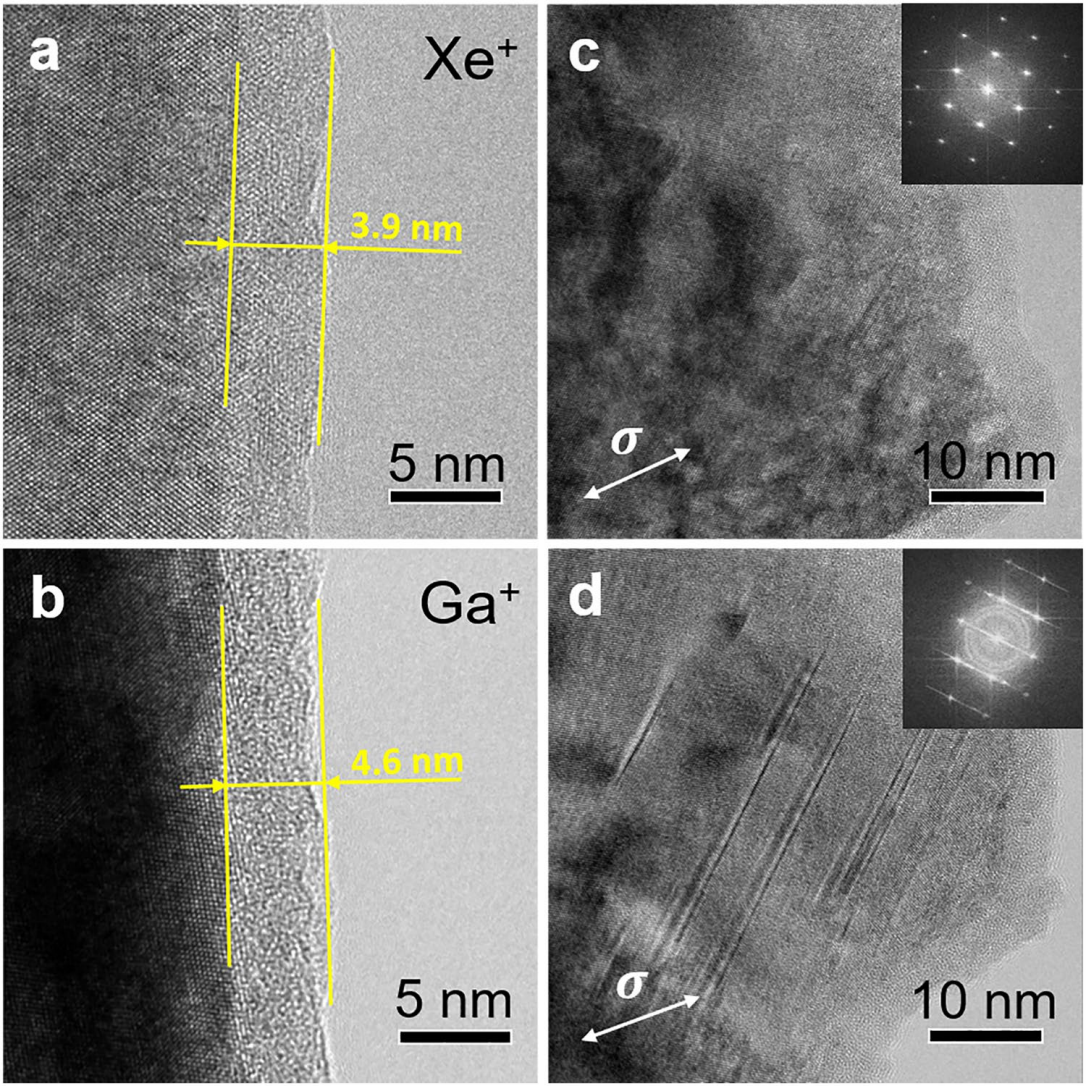
TEM images of Xe^+^ fabricated (first row) and Ga^+^ fabricated (second row) samples. (**a,b**) The sample surface before tensile deformation showing the thickness of the amorphous layer. (**c,d**) Fracture regions of the two samples after tensile deformation. The double arrows in (**c**,**d**) indicate the loading direction.

Representative microstructures before and after fracture for the two types of samples are presented in the TEM images in Fig. 3. Both sample preparation techniques resulted in an amorphous layer at the surface, which is a common phenomenon for materials processed by FIB milling^30,31,61^. For thickness measurement of the amorphous layers, the samples were oriented horizontally in the TEM to ensure the measured thickness was comparable. The thickness of the amorphous layers was ~3.9 nm (Fig. 3a) and ~4.6 nm (Fig. 3b) for the Xe^+^ fabricated and the Ga^+^ fabricated samples, respectively. Since the gauge widths of the samples were ~300 nm, the volume fraction of the amorphous layer at the gauge region was ~2.6% and ~3.0% for the Xe^+^ fabricated and the Ga^+^ fabricated samples, respectively. As a result of the minor variation in the volume fractions, the amorphous layers likely contribute little to the distinction of the mechanical properties between the two sample groups.

In the region immediately beneath the amorphous layer, where ion implantation damage typically occurs^30^, a high density of nanotwins and stacking faults (SFs) can be observed in deformed Ga^+^ fabricated samples (Fig. 3d), but not in deformed Xe^+^ fabricated samples (Fig. 3c). Formation of a high density of nanotwins/SFs (the average twin/SF spacing in Fig. 3d was only 4.3 nm) strengthens materials but also makes them brittle^62^.

Major factors that affect the propensity of deformation twinning include crystal structure, grain size (or sample size for single crystals), crystallographic orientation, strain rate, temperature, applied stress, and stacking fault energy (SFE)^63^. As the two types of samples have the same sample size, the same crystallographic orientation (relative to the stressing direction), and the same deformation conditions, the only two factors that could have led to the significant variation of the deformation twinning propensity are the applied stress and SFE of the samples. Higher applied stress usually benefits the activation of deformation twinning^63^ which in turn leads to further strengthening. Alloying of the material caused by Ga^+^ implantation could alter the SFE of the HEA, which has been reported for other alloying elements, and therefore promote deformation twinning^64,65^. Conversely, Xe^+^ plasma introduces an insignificant alloying effect since Xe^+^ is an inert gas.

**Figure 4:**
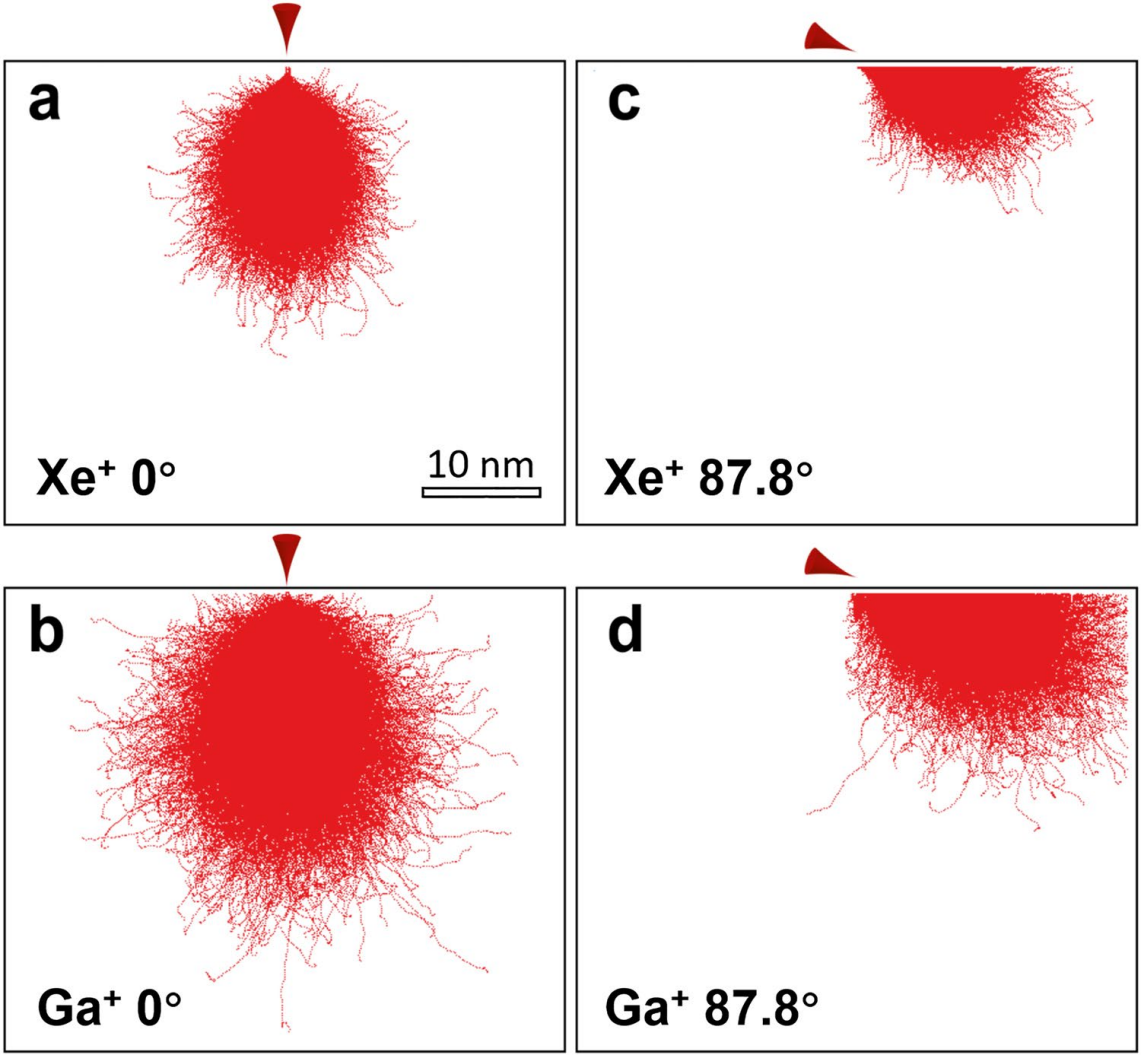
The SRIM^66^ predictions for ion trajectories showing the penetration depth of Xe^+^ and Ga^+^ into the CrMnFeCoNi high-entropy alloy at an accelerating voltage of 30 kV. (**a,b**) Xe^+^ PFIB and Ga^+^ FIB, respectively, with the normal incidence angle (0°). (**c,d**) Xe^+^ PFIB and Ga^+^ FIB, respectively, with a grazing incidence angle of 87.8°.

To estimate the depth of the damaged zone resulting from ion implantation, Monte Carlo simulations were carried out using the SRIM software package^66^ to calculate the trajectories of Xe^+^ and Ga^+^ implanted into the CrMnFeCoNi HEA. While SRIM calculations present the sum of single events that do not include dynamic developments caused by effects of neighbouring atoms, composition changes by preferential sputtering and implantation and deposition of the FIB projectiles^66^, they are good enough to explain the effects of different types of ion sources on the depth of the damaged zone. Other simulation methods, such as the TRI3DYN code^67^, are available for a more comprehensive outcome. The results of SRIM calculations are shown in Fig. 4a–d. Incident angles of 0° and 87.8° were utilized in the simulations to imitate the experimental conditions. Other parameters used in the simulations include the ion energy, which was set to 30 keV for consistency with experimental conditions, as well as the total number of ions, which was set to 20,000 to permit adequate calculation. In both situations, Xe^+^ introduced a much smaller affected volume (depth of ion range ~25 nm at normal incidence angle and ~13 nm at 87.8°) than Ga^+^ (depth of ion range ~38 nm at normal incidence angle and ~20 nm at 87.8°). This affected volume consists of two parts: the amorphous structure in the surface layer and the damaged zone immediately beneath the amorphous layer. Since the damaged zone, which is rich in dislocations and point defects, hardens materials^30^, it is reasonable to believe that Ga^+^ FIB milling leads to a more significant hardening effect than Xe^+^ PFIB milling but that it also lowers the ductility.

## Conclusions

Comprehensive investigation and comparison of the effects of Xe^+^ PFIB and Ga^+^ FIB on the microstructure and mechanical properties of a high-entropy alloy have been carried out using *in situ* tensile straining TEM, detailed microstructural characterisation and Monte Carlo simulations. The results indicate that samples prepared with Xe^+^ PFIB present improved ductility but less strength than those prepared with Ga^+^ FIB. This is because the Xe^+^ PFIB processing produces a smaller damaged zone immediately beneath the amorphous layer, and exerts an insignificant alloying effect to materials. In addition, the introduction of Ga^+^ to the high-entropy alloy during the FIB milling process results in a locking effect on dislocation motion and a reduction of the SFE of the material. Both factors result in a strengthening of the materials but also embrittle it, while such effects are considerably absent in Xe^+^ prepared samples. Together with the fact that Xe^+^ PFIB is several times more efficient in material removal rate than Ga^+^ FIB, the Xe^+^ PFIB technique clearly demonstrates a superb alternative for specimen preparation for nanomechanical experiments dealing with HEAs. Further investigation is needed to validate the superiority of Xe^+^ PFIB in other material systems. On the other hand, Ga^+^ FIB can be used to prepare small-sized specimens when a further strengthening of materials is essential.

## Supplementary information

Supplementary Movie S1.

Supplementary Movie S2.

Supplementary Movie S3.

Supplementary Movie S4.

Supplementary Information.

## Data Availability

*In situ* tensile straining TEM videos for sample Ga#1, Ga#2, Xe#1 and Xe#2 are available in the supplementary information. All other data included in this study are available upon request by contact with the corresponding author.
