# Supplementary material for: Effect of Ion Irradiation Introduced by Focused Ion-Beam Milling on the Mechanical Behaviour of Sub-Micron-Sized Samples
Source: Sci Rep. 2020 Jun 25;10:10324. doi: 10.1038/s41598-020-66564-y (PMC7316792; doi:10.1038/s41598-020-66564-y)
Supplement: Supplementary file 5 — Supplementary Information. [file 41598_2020_66564_MOESM5_ESM.docx]

**Effect of Ion Irradiation Introduced by Focused Ion-Beam Milling on the Mechanical Behaviour of Sub-Micron-Sized Samples**

Jinqiao Liu^1^, Ranming Niu^1,^*, Ji Gu^2^, Matthew Cabral^1^, Min Song^2^ , Xiaozhou Liao^1^

^1^ School of Aerospace, Mechanical & Mechatronic Engineering, The University of Sydney, Sydney, NSW 2006, Australia

^2^ State Key Laboratory of Powder Metallurgy, Central South University, Changsha, 410083, China

*Correspondence and requests for materials should be addressed to R.Niu (e-mail: ranming.niu@sydney.edu.au)

Supplementary Information

**Movie 1 | In-situ tensile straining TEM video of sample Ga#1.** The sample is loaded along <001> orientation, with a controlled strain rate of 10^-3^ s^-1^. The zone axis is near <110> orientation. The video clip plays at 5 × speed.

**Movie 2 | In-situ tensile straining TEM video of sample Ga#2.** The sample is loaded along <001> orientation, with a controlled strain rate of 10^-3^ s^-1^. The zone axis is near <110> orientation. The video clip plays at 5 × speed.

**Movie 3 | In-situ tensile straining TEM video of sample Xe#1.** The sample is loaded along <001> orientation, with a controlled strain rate of 10^-3^ s^-1^. The zone axis is near <110> orientation. The video clip plays at 5 × speed.

**Movie 4 | In-situ tensile straining TEM video of sample Xe#2.** The sample is loaded along <001> orientation, with a controlled strain rate of 10^-3^ s^-1^. The zone axis is near <110> orientation. The video clip plays at 5 × speed.
